# Correspondence: Challenges with dating weathering products to unravel ancient landscapes

**DOI:** 10.1038/s41467-017-01457-9

**Published:** 2017-11-15

**Authors:** Haakon Fossen, Anna K. Ksienzyk, Joachim Jacobs

**Affiliations:** 1 Museum of Natural History/Department of Earth Science, University of Bergen, Allégaten 41, N-5007 Bergen, Norway; 2 Department of Earth Science, University of Bergen, Allégaten 41, N-5007 Bergen, Norway; 3 Norwegian Polar Institute, Fram Centre, 9296 Tromsø, Norway

K-Ar dating of illite (clay) in weathered bedrock (saprolite) is an exciting but yet incompletely understood new application of the K-Ar dating method that can potentially provide valuable information about the evolution of landforms and continental isostasy. Fredin et al.^1^ use this approach in an attempt to date the strandflat in coastal western Scandinavia. Based on K-Ar illite ages from three widely separated localities in the North Sea (Utsira High), West Norway (Bømlo), and southern Sweden (Ivö), they suggest a Late Triassic (~210 Ma) age for the strandflat. However, when employing such a new methodology, it is particularly important to carefully consider the results together with existing data, and Fredin et al.^1^ neglect previously published radiometric, stratigraphic, and geomorphic constraints that strongly suggest that the current strandflat erosional level in western Norway is younger than Triassic.

**Fig. 1:**
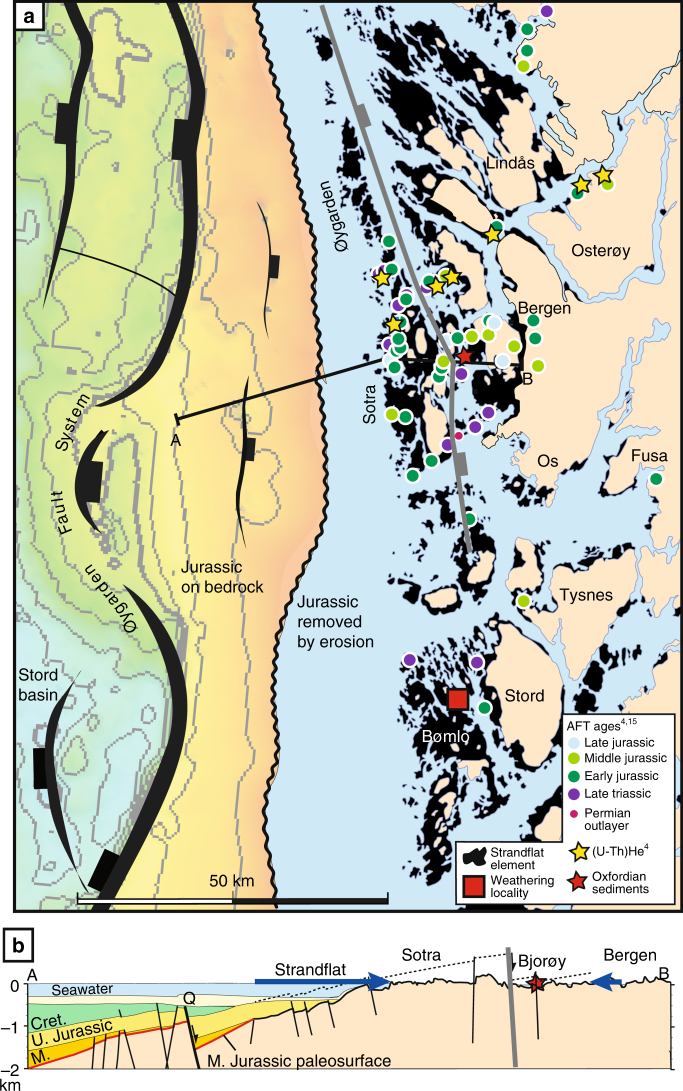
Map of and cross-section through the strandflat. **a** Map of the strandflat area in SW Norway, and offshore fault and top basement map. The erosion line marks the eastern boundary of Jurassic sediments on basement. Red square marks sampling locality by Fredin et al.^1^ (Bømlo). AFT localities (from Ksienzyk et al.^4^ and Kohlman et al.^15^) are color-coded with respect to age. **b** Cross-section^2^, showing the west-dipping Jurassic paleosurface buried under Middle and Late Jurassic sediments and cut by the strandflat near sea level

The discovery of Late Jurassic (Oxfordian) sediment caught up in a fault zone in Proterozoic bedrock near Bergen north of Bømlo (Fig. 1) revealed that rocks in the strandflat area were at or near the surface at ~160 Ma^2^, opening the possibility that the strandflat may contain Mesozoic elements^3^. Offshore, the crystalline bedrock surface is seen as a remarkably planar geomorphic feature on seismic data, preserved under Jurassic sediments (offshore part of Fig. 1b). However, this surface is dipping to the west by ~5°, while the strandflat is almost horizontal (onshore part of Fig. 1b; also shown in Fig. 6 in Fredin et al.^1^), clearly cutting into the Middle Jurassic paleosurface and thus mainly shaped by younger (post-Middle Jurassic) processes. From geometric considerations, it is therefore quite unlikely that the samples from the Utsira High and Bømlo represent the same weathering surface.

Fredin et al.^1^ claim to be able to constrain the age of the strandflat along the west coast of Norway by dating illite in weathered bedrock. However, K-Ar dating of illite to constrain weathering ages is previously untested; all previous studies cited by Fredin et al.^1^ use K-bearing manganese oxides or alunite-group sulfates. Hence, such K-Ar illite weathering ages should be interpreted with care and in the framework of independent data, which in this case include low-temperature thermochronology (fission track and (U–Th)/He ages), the offshore stratigraphic record, structural aspects, and the estimated depth of dike intrusions, as briefly summarized below.

A significant quantity of fission track and (U–Th)/He data has recently been published from the strandflat area^4^. Such ages date the cooling of the currently exposed rocks through the partial annealing/retention zone of the respective system, which is 210–140 °C for the zircon (U–Th)/He system, 120–60 °C for the apatite fission track (AFT) system and 70–40 °C for the apatite (U–Th)/He system. All such ages should be older than the age of any preserved in situ weathering products. A regional compilation of AFT ages from Scandinavia^5^ shows that AFT ages from the entire Norwegian strandflat area are similar to or, more commonly, younger than the ~210 Ma illite ages reported by Fredin et al^1^. Most ages from the strandflat region relatively near their Bømlo locality show early to middle Jurassic (200–160 Ma) AFT ages^4^ (Fig. 1a). These ages roughly indicate that the samples were buried at >2 km depth in the Early Jurassic, assuming a thermal gradient of 30 °C/km. (U–Th)/He zircon data from the same area of ~225 Ma^4^ suggest burial of the present strandflat level to >4 km depth in the Late Triassic. These data are consistent with paleomagnetic analysis of Permian (~250 Ma) dikes in the strandflat area north of Bømlo, which suggests that the dikes were emplaced at ambient temperatures between 150–500 °C (5–15 km depth)^6^.

**Fig. 2:**
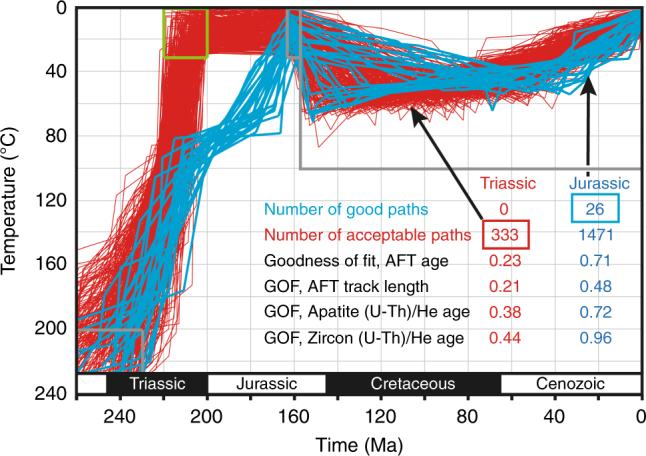
Time-temperature paths. The paths are derived from AFT and apatite and zircon (U-Th)/He data (sample BG-113 in Ksienzyk et al.^4^). Red lines are 333 acceptable-fit paths, where the sample was forced to the surface in the Late Triassic and kept there until the Late Jurassic. Blue lines are good-fit paths, where the sample was brought to the surface in the Late Jurassic (the 1471 acceptable paths for this model are not shown). The latter model is favored because it produces many more acceptable paths and also many good paths, while the first case (red) produced no good paths. Gray boxes indicate constraints for both models, while green box applies only to Triassic surfacing (red) model

In slowly cooled basement terranes like western Norway, it can be misleading to reconstruct the exhumation history based on fission track ages alone. More precise and detailed cooling paths can be derived from inverse time–temperature modeling. The resulting models, presented by Ksienzyk et al.^4^, consistently show cooling throughout the Triassic and into the Jurassic, with post-Jurassic burial and new exhumation for coastal samples (Fig. 2, blue curves). In order to test for potential Late Triassic weathering, we remodeled strandflat samples by imposing constraints to bring them to the surface in the Late Triassic (green box in Fig. 2). With this constraint, most models showed a significantly reduced fit with the data. More specifically, cooling paths with a good fit that are supported by the data (good paths) were not obtainable, only paths with a lower fit that are merely “not ruled out by the data”^7^ (so-called “acceptable paths”; red curves in Fig. 2). Furthermore, those acceptable paths involve unrealistically rapid cooling, implying almost instantaneous exhumation from ~3 km depth to the surface around 220 Ma (Fig. 2, red curves). Thus, the present thermochronologic data set does not support a Late Triassic weathering scenario.

Looking at the stratigraphic record, the offshore Jurassic basement paleosurface is abruptly offset by the major North Sea rift-bounding Øygarden Fault System, which bounds the Stord basin and it is up to 4–5 km of Triassic-Jurassic clastic sediments to the west^8^ (Fig. 6 in Fredin et al.^1^). A significant part of these sediments is late Triassic-Jurassic, and the basin geometry suggests a proximal onshore source^9^. Hence, removal of considerable amounts of bedrock in the coastal area of SW Norway through the Triassic-Jurassic, as suggested by low-temperature thermochronologic data referred to above, is consistent with the offshore stratigraphic record.

Finally, faulting in the strandflat region and immediately offshore SW Norway occurred over a long time period, and includes late Jurassic-early Cretaceous activity^10,11^ with local offsets of up to several hundred meters^12^ (Fig. 1). However, the strandflat is not affected by such offsets, suggesting that its formation or completion occurred after the late Jurassic.

In summary, Fredin et al.’s K-Ar illite dates and their implications for landscape evolution in western Scandinavia should be reconsidered in the light of independent constraints, which consistently show that the strandflat is unlikely to be as old as Triassic. We do not attempt to reinterpret their isotopic data here, but raise the question whether their Triassic illites at the Bømlo locality may have grown in a subsurface fracture system prior to exhumation, as reported recently for a close-by locality by the same research group^13^. As for the offshore Utsira data presented by Fredin et al.^1^, they are in agreement with recent zircon (U-Th)/He, AFT and apatite (U-Th)/He dating that shows that the basement surface in that particular structural high reached near-surface temperatures in Carboniferous-Triassic times^14^. However, there is no reason to believe that these two surfaces should be of the same age, as the top basement surface in the northern North Sea basin is well known to be diachronous throughout the basin. We believe that the interesting post-Caledonian history of western Scandinavia can be understood only through an integrated effort that takes all available data into account, and urge the authors to critically reconsider their interpretations accordingly.
